# Spatial Analysis of Hemorrhagic Fever with Renal Syndrome in Zibo City, China, 2009–2012

**DOI:** 10.1371/journal.pone.0067490

**Published:** 2013-06-28

**Authors:** Feng Cui, Tao Wang, Ling Wang, Shuxia Yang, Ling Zhang, Haixia Cao, Yan Zhang, Haodong Hu, Shenyong Zhai

**Affiliations:** Zibo Municipal Center for Disease Control and Prevention, Zibo, Shandong Province, People’s Republic of China; National Institutes of Health. National Institute of Allergy and Infectious Diseases, Division of Clinical Research, United States of America

## Abstract

**Background:**

Hemorrhagic fever with renal syndrome (HFRS) is highly endemic in mainland China, where human cases account for 90% of the total global cases. Zibo City is one of the most serious affected areas in Shandong Province China with the HFRS incidence increasing sharply from 2009 to 2012. However, the hotspots of HFRS in Zibo remained unclear. Thus, a spatial analysis was conducted with the aim to explore the spatial, spatial-temporal and seasonal patterns of HFRS in Zibo from 2009 to 2012, and to provide guidance for formulating regional prevention and control strategies.

**Methods:**

The study was based on the reported cases of HFRS from the National Notifiable Disease Surveillance System. Annualized incidence maps and seasonal incidence maps were produced to analyze the spatial and seasonal distribution of HFRS in Zibo City. Then spatial scan statistics and space-time scan statistics were conducted to identify clusters of HFRS.

**Results:**

There were 200 cases reported in Zibo City during the 4-year study period. One most likely cluster and one secondary cluster for high incidence of HFRS were identified by the space-time analysis. And the most likely cluster was found to exist at Yiyuan County in October to December 2012. The human infections in the fall and winter reflected a seasonal characteristic pattern of Hantaan virus (HTNV) transmission. The secondary cluster was detected at the center of Zibo in May to June 2009, presenting a seasonal characteristic of Seoul virus (SEOV) transmission.

**Conclusion:**

To control and prevent HFRS in Zibo city, the comprehensive preventive strategy should be implemented in the southern areas of Zibo in autumn and in the northern areas of Zibo in spring.

## Introduction

Hemorrhagic fever with renal syndrome (HFRS), a rodent-borne disease caused by hantaviruses (family Bunyaviridae), is characterized by fever, acute renal dysfunction, and hemorrhage manifestations [1], [2]. Various rodent species are natural hosts and serve as sources of infection [3]. In China, the causative agents of HFRS are predominately Hantaan virus (HTNV) and Seoul virus (SEOV), which result in case fatality rates of approximately 10% and 1%, respectively [4]. HTNV causes a more severe form of HFRS than SEOV does and is associated with *Apodemus agrarius* (striped field mouse), while SEOV is typically carried by *Rattus norvegicus* (Norway rat) [3], [5]–[7]. Occurrence of HFRS cases is seasonal with a bimodal pattern and studies suggest that the pattern is linked to varying transmission dynamics of the two serotypes of hantaviruses among their animal hosts. HTNV-caused HFRS cases occur year-round but tend to peak in the winter while SEOV-caused infections typically peak in the spring [6], [8], [9]. Hantaviruses are primarily transmitted from rodent host to human by aerosols generated from contaminated urine and feces and possibly from contaminated food or rodent bites [10]–[12].

HFRS was first recognized in northeastern China in 1931 and has been prevalent in many other parts of China since 1955. At present, it is highly endemic in mainland China accounting for 90% of the total cases reported in the world [10], [12]–[14]. Several studies showed that the spatial distribution of HFRS was nonrandom and clustered, such as Beijing [3], , Liaoning [15], [16], Shandong [6], [17], Shenyang [18]. Historically, Shandong Province bears the largest HFRS burden in China, the cumulative human cases accounted for 1/3 of the national total [6]. And Zibo City is one of the most serious affected areas in Shandong Province China [6]. The HFRS incidence has been increasing in Zibo City in recent years according to the report from Zibo Center for Disease Control and Prevention (CDC). However, the hotspots of HFRS in Zibo remained unclear. A better understanding of the spatial distribution patterns of HFRS would help to identify areas and population at high risk, and might help health departments to provide guidance for formulating regional prevention and control strategies.

Therefore, we conducted GIS-based spatial and space-time scan statistics analysis to characterize the spatial, spatial-temporal and seasonal patterns of HFRS at town-level in Zibo City of China during 2009–2012. The conclusions of our study will be essential for determining the approach to rodent control or to vaccination for HFRS prevention and control on a small geographic scale.

## Materials and Methods

### Study Area

**Figure 1:**
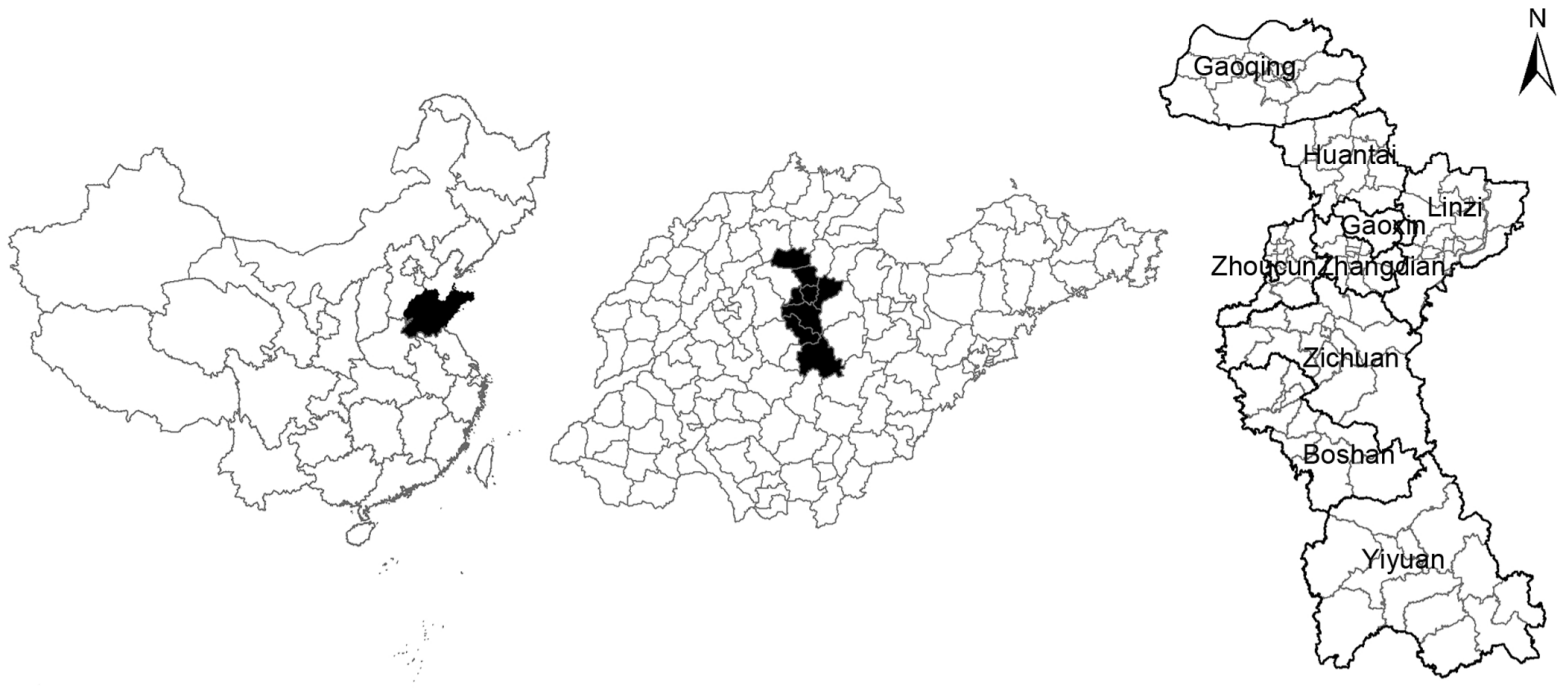
Location of the study area, Zibo City in Shandong Province, China.

The study site is located in Zibo City (latitude 35°55′∼37°17′ N and longitude 117°32′∼118°31′ E), in the central part of Shandong province (Figure 1). The city includes 88 towns belonging to 9 counties with a total land area of 5 965 square kilometers and a population of about 4.22 million. It has a temperate and monsoonal climate with four clearly distinct seasons. In Zibo City, the southern areas are mountainous and hilly, and the northern areas are plain.

### Data Collection and Management

The data on reported HFRS cases including information about sex, age, residential address, and onset date of symptoms for each case were obtained from the National Notifiable Disease Surveillance System, and we were permitted to use the data. To conduct a GIS-based analysis on the spatial distribution of HFRS, the town-level polygon map at 1∶100 000 scale was obtained, on which the town-level point layers containing information regarding latitudes and longitudes of central points of each town were created. Demographic information based on Zibo Statistical Yearbook was integrated in terms of the administrative code. All HFRS cases were geo-coded and matched to the town-level layers of polygon and point by administrative code using the software ArcGIS9.3 (ESRI Inc., Redlands, CA, USA).

### Demographic Distribution Analysis

The demographic distribution characteristics including age, sex and occupation distribution of HFRS cases from 2009 to 2012 in Zibo City were analyzed according to surveillance data.

### Geographical Information System (GIS) Mapping for Incidence of HFRS

Based on annual incidence, all towns were grouped into four categories [5], [19]: non-endemic areas, low endemic areas with an annual incidence between 0 and 5 per 100 000, medium endemic areas with an incidence between 5 and 15 per 100 000, and high endemic areas with an incidence over 15 per 100 000. The four types of counties were color-coded on the maps.

To alleviate variations of incidence in small populations and areas, annualized average incidence of HFRS per 100 000 at each town over the 4 year-period were calculated. Furthermore, annualized average incidences and the proportion of monthly average incidence for each town were mapped in gradient colors and pie charts, respectively.

### Spatial Cluster Analysis

Spatial and space-time scan statistics [20]–[24] were applied to identify clusters of HFRS. The analyses were performed each given year using SaTScan™ v9.1.1 software (http://www.satscan.org/). The null hypothesis assumed that the relative risk (RR) of HFRS was the same within the window compared to outside [19]. In this analysis, a Poisson based model was used, where the number of events in an area is Poisson distributed according to a known underlying population at risk [25]. The spatial scan statistic imposes a circular (or elliptic) window which is in turn centered on each geographical area throughout the study region. The radius of the window varies continuously in size from zero to some upper limit specified by the user. The space-time scan statistic is defined by a cylindrical window with a circular (or elliptic) geographic base and with height corresponding to time [21].

For this study, in order to find possible sub-clusters, the maximum spatial cluster size was set as 10% of the total population at risk, and the maximum temporal cluster size was set as 50% of the total study period. The test of significance of the identified clusters was based on comparing the likelihood ratio test statistics against a null distribution obtained from Monte Carlo Simulation [24], [26]. The number of permutation was set to 999 and the significance level was set as 0.05.

## Results

### Descriptive Analysis of HFRS in Zibo City

**Figure 2:**
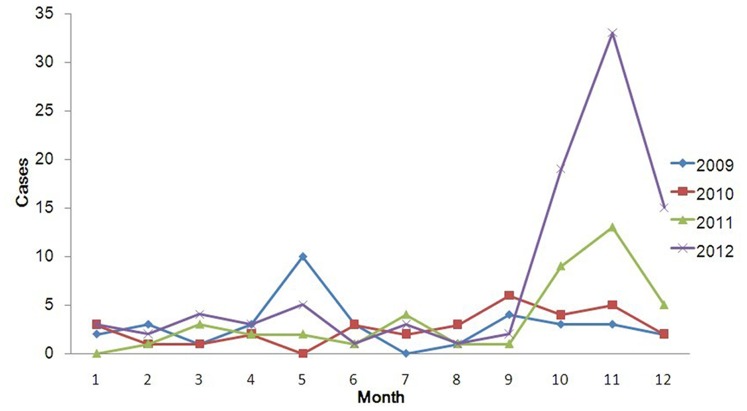
Monthly distribution of HFRS cases, 2009–2012.

A total of 200 cases were reported in Zibo City during the 4-year study period. Of these, 76% were male and 24% were female, with the sex ratio (male vs. female) 3.17. Among these patients, 3% were in children ≤14 years of age, 89% were in persons 15–64 years of age, and 8% were in persons ≥65 years of age. Regarding to occupation, 68% of HFRS patients were farmers, 10% were workers (mainly forestry workers, builders), and followed by students which accounted for 6%. Poor housing conditions and high rodent density in rural areas seem to be responsible for most HFRS epidemics. The monthly distribution of HFRS cases was shown in figure 2, which indicated that the occurrence of HFRS presented significant seasonality.

**Figure 3:**
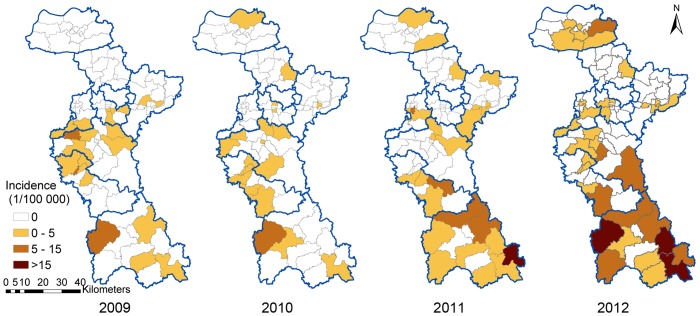
The HFRS incidence rate (/10 000) per year and per town.

**Figure 4:**
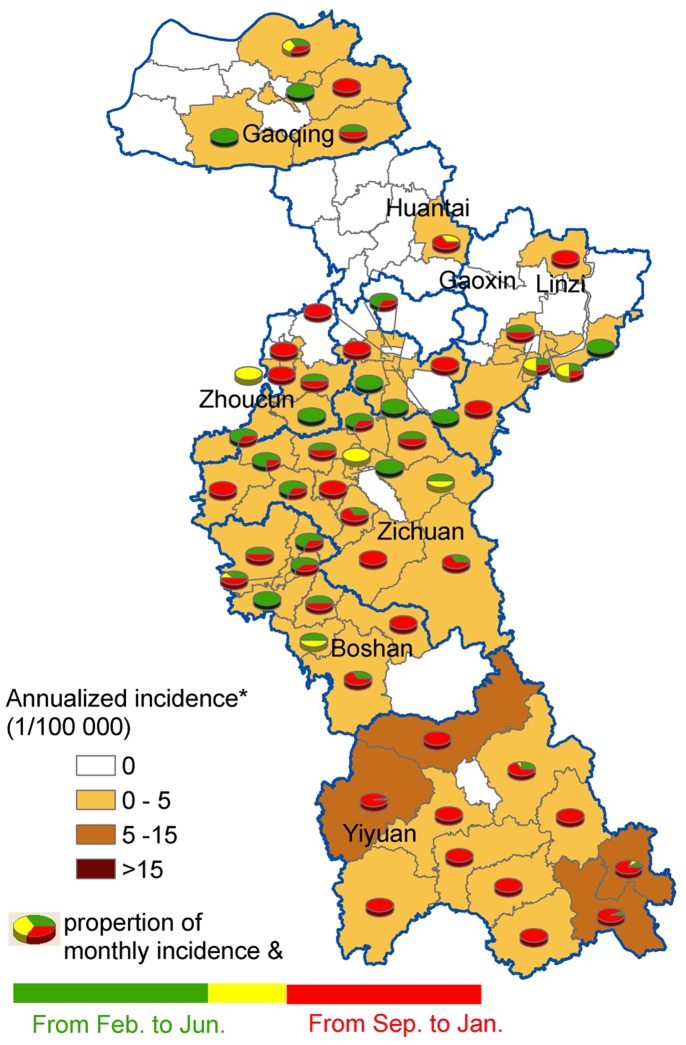
The spatial distribution of HFRS incidence and their proportion of monthly incidence in each town. The background of map with color gradient presents the annual incidence of HFRS, and pie graphs display the proportion of monthly incidence for each town. Towns in white on the map have zero incidences. * Average annual incidence per 100 000 populations. & Proportion of average monthly incidence in these pie graphs, where green color indicates the proportion of average monthly incidence from February to June (in spring and early summer), yellow is the proportion of average monthly incidence from July to August (in summer), and the red represents the proportion of average monthly incidence from September to January (in autumn and winter).

The HFRS incidence rates per year and per town were summarized in Figure 3. The annual incidence had sharply increased from 2009 to 2012. Figure 4 showed annualized average incidence and the proportion of monthly average incidence for each town. Annualized average incidence at the town-level ranged from 0 to 10.1 per 100 000.Among the total 88 towns in Zibo, 31 towns were non-endemic, 54 towns were low-endemic, and 4 towns were medium-endemic. The main endemic areas of HFRS were located in the south of Zibo City with a single epidemic peak in the fall-winter season mapped by the red color of the pies. In north Zibo City, an epidemic peak in the spring season was mapped by the green color in the pies (Figure 4).

### Purely Spatial Analysis

**Figure 5:**
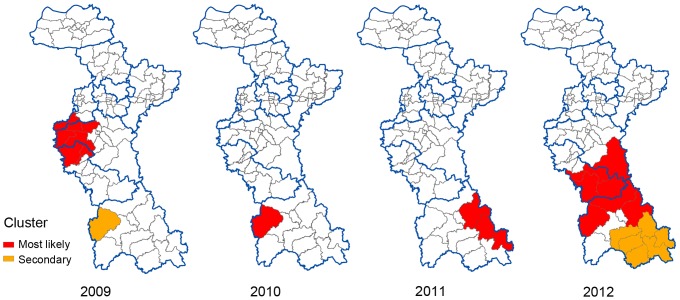
Spatial clusters of HFRS in Zibo City, China from 2009 to 2012.

For the purely spatial analyses, the clusters were detected for each year from 2009 to 2012. The significant clusters of a high incidence of HFRS were listed in Table 1, and depicted on the map in Figure 5, which indicated that the locations and sizes of these clusters varied by year.

**Table 1:** SaTScan statistics for spatial clusters with significant higher incidence in Zibo City, China from 2009 to 2012.

Year	Cluster Type	Location	Observed Cases	Expected Cases	Relative Risk	*P*-value
2009	Most likely	Shangjia, Wangcun, Zhonglou, Lingzi,Kunlun, Yucheng, Baita, Chengxi	14	3.35	6.40	<0.001
	Secondary	Lucun	5	0.62	9.21	0.045
2010	Most likely	Lucun	7	0.59	14.96	<0.001
2011	Most likely	Yuezhuang, Shiqiao, Zhangjiapo	13	1.19	15.22	<0.001
2012	Most likely	Lucun, Nanlushan, Yuezhuang, Taihe, Yuanquan,Shima, Boshan, Chishang	37	7.70	7.46	<0.001
	Secondary	Shiqiao, Zhangjiapo, Yanya, Dongli, Xili,Zhongzhuang	19	4.63	4.94	<0.001

### Space-time Analysis of HFRS in Zibo City

**Figure 6:**
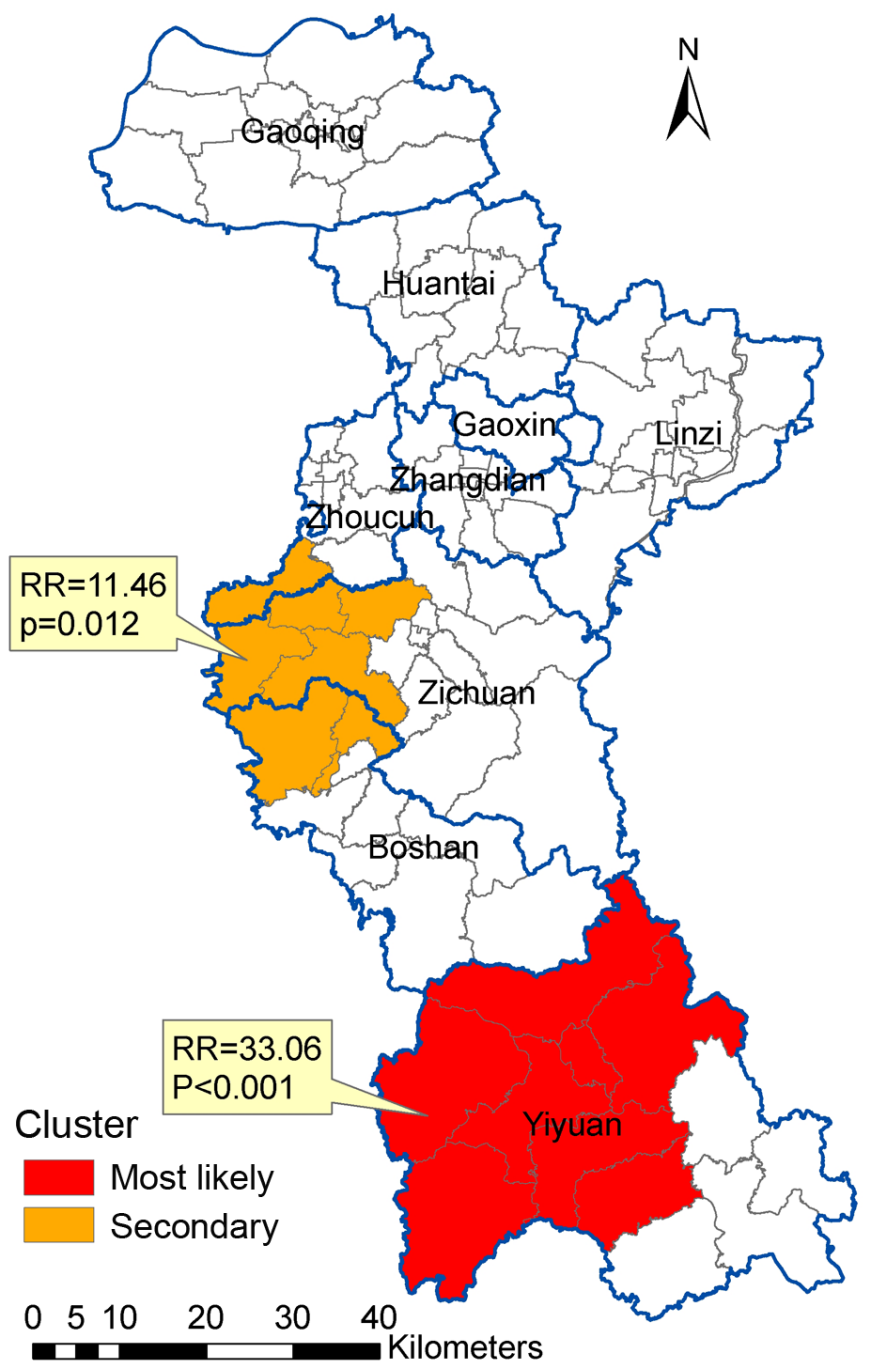
Space-time clusters of HFRS in Zibo City, China from 2009 to 2012.

Space-time cluster analysis of HFRS in 2009–2012 in Zibo City revealed that HFRS was not distributed randomly in space-time (Table 2 and Figure 6). The most likely statistically significant cluster for high incidence of HFRS was found at Yiyuan County in October to December 2012 (RR = 33.06, p<0.001), with 33 observed cases and 1.19 expected cases. One statistically significant secondary cluster was also detected for high incidence of HFRS, which had 9 observed cases and 0.82 expected cases (RR = 11.46, p = 0.012) in May to June 2009.

**Table 2:** SaTScan statistics for space-time clusters with significant higher incidence in Zibo City, China from 2009 to 2012.

Cluster Type	Time Frame	Location	ObservedCases	ExpectedCases	RelativeRisk	*P*-value
Most likely	2012/10/1 to 2012/12/31	Lucun, Nanlushan, Yuezhuang, Yanya, Dazhuangzhuang, Zhongzhuang, Nanma	33	1.19	33.06	<0.001
Secondary	2009/5/1 to 2009/6/30	Shangjia, Wangcun, Zhonglou, Lingzi, Kunlun, Yucheng, Baita, Chengxi	9	0.82	11.46	0.012

## Discussion

In this study, our results demonstrated that the HFRS incidence had been increasing from 2009 to 2012 and the main re-emerging endemic areas of HFRS were located in the south of Zibo City. The proportion of monthly average incidence for each town showed that HFRS in the southern region of Zibo was mainly caused by HTNV, and in the northern was mainly caused by SEOV. For the purely spatial analyses, the clusters were detected for each year from 2009 to 2012. One most likely cluster and one secondary cluster for high incidence of HFRS were identified by the space-time analysis.

In epidemiology, cluster analyses are of importance in detecting aggregation of disease cases, testing the occurrence of any statistically significant clusters, and ultimately finding evidences of etiologic factors. Recently, scan statistic implemented in SaTScanTM software is being widely used to detect and evaluate clusters for a variety of diseases, including cancer [26]–[28], HFRS [3], [16], tuberculosis [29]–[31], Creutzfeldt-Jakob disease [32], etc. In our study, the clusters detected by the purely spatial analyses provided reference of quantitative measurements for the geographic areas of high HFRS risk.

Space-time scan statistic method is proposed as a dynamic supplement to purely spatial statistical methods for outbreak detection and prediction. One most likely cluster and one secondary cluster for high incidence of HFRS were identified by the space-time analysis. The most likely cluster was found at Yiyuan County in October to December 2012, and the secondary cluster was detected at the centers of Zibo City in May to June 2009. Previous studies reported that the transmission of HTNV through *Apodemus agrarius* peaked in the winter, while *Rattus norvegicus* associated SEOV infections mainly occurred in the spring [6], [8], [9], [33]. Thus, the human infections in the most likely cluster in the fall and winter reflect a seasonal characteristic pattern of HTNV transmission, and a seasonal characteristic of SEOV transmission in the secondary cluster. The HFRS endemic areas have spread from the initial center of Zibo in 2009 towards the southern parts of Zibo in 2012 with shifts of causal agents of HFRS – SEOV to HTNV. The dominant rodent species might also have shifted from *Rattus norvegicus* to *Apodemus agrarius*, and further rodent HFRS surveillance, including using virus isolation method to discriminate HFRS cases due to different hantaviruses, should be enhanced.

Yiyuan, the most likely cluster, and Zichuan, the secondary cluster are mountainous and hilly with numerous *Apodemus agrarius*, and farmers have more chances to be exposed to contaminated urine and feces of infected rodents compared to neighboring localities. Agricultural activities such as sleeping in the fields, irrigating, and working on the farmland during the autumn harvest season might have played a significant role in the occurrence of HFRS [34]. Therefore, for the southern areas of Zibo city where the HFRS cases manily caused by HTNV, deratization in wild areas should be enhanced in the late autumn and early winter; for the northern areas of Zibo city where the HFRS cases mainly caused by SEOV, derationzation in residential areas and reducing human exposure to infected rodents and their excrements should be enhanced in spring.

The application of GIS, together with spatial statistical techniques, provides ways to quantify explicit HFRS and to further identify environmental factors responsible for the increasing disease risk. Although analyses are still preliminary, the findings can be helpful for generating hypothesis for further investigation. Future researches are warranted to focus on the risk factors of HFRS such as rodent population densities, human activities, farming patterns, various socio-economic and environmental factors in the clustering areas including Yiyuan and Zichuan county. Despite insights gained from our study, the limitations of our study should also be acknowledged. Firstly, due to a lack of time series data on the Hantaviruses of HFRS cases, population densities of rodents, and the influencing factors, it is difficult to further uncover the probable causes of the characters and shifts of the spatial, spatial-temporal and seasonal patterns of HFRS. Secondly, our data are from a passive surveillance system, and cases might be underreported, which then might influence the precision of our analyses.

In summary, the result of our study provide useful information on the prevailing epidemiological situation of HFRS in Zibo City. The novel knowledge about the presence of clusters of HFRS in Zibo can help the Zibo CDC to intensify their remedial measures in the identified areas of high HFRS prevalence and chalk out future strategies for more effective HFRS control. To control and prevent HFRS, a comprehensive preventive strategy including public health education and promotion, rodent control, surveillance, and vaccination should be implemented in the southern areas of Zibo in autumn and in the northern areas of Zibo in spring.
